# LDL receptor in alphavirus entry: structural analysis and implications for antiviral therapy

**DOI:** 10.1038/s41467-024-49301-1

**Published:** 2024-06-08

**Authors:** Ningning Wang, Andres Merits, Michael Veit, Laura Sandra Lello, Shuhan Kong, Houqi Jiao, Jie Chen, Yu Wang, Georgi Dobrikov, Félix A. Rey, Shuo Su

**Affiliations:** 1 Engineering Laboratory of Animal Immunity of Jiangsu Province, College of Veterinary Medicine, Academy for Advanced Interdisciplinary Studies, Nanjing Agricultural University, Nanjing, China; 2 Institute of Bioengineering, University of Tartu, Nooruse Street 1, Tartu, Estonia; 3 Institute for Virology, Center for Infection Medicine, Veterinary Faculty, Free University Berlin, Berlin, Germany; 4 Institute of Organic Chemistry with Centre of Phytochemistry, Bulgarian Academy of Sciences, Acad. G. Bonchev Street, Bl. 9, Sofia, Bulgaria; 5 Institut Pasteur, Unité de Virologie Structurale, Department Virologie, CNRS UMR 3569, 25-28 Rue du Docteur Roux, 75724 Paris Cedex 15, Paris, France; 6 Sanya Institute of Nanjing Agricultural University, Nanjing Agricultural University, Sanya, China

**Keywords:** Alphaviruses, Virus structures

## Abstract

Various low-density lipoprotein receptors (LPRs) have been identified as entry factors for alphaviruses, and structures of the corresponding virion-receptor complexes have been determined. Here, we analyze the similarities and differences in the receptor binding modes of multiple alphaviruses to understand their ability to infect a wide range of hosts. We further discuss the challenges associated with the development of broad-spectrum treatment strategies against a diverse range of alphaviruses.

## Abstract (web summary)

Recently, studies have identified different low-density lipoprotein receptors as functional entry receptors for alphaviruses. In this Perspective, the authors compare the known alphavirus-receptor structures and discuss why it might be challenging to generate a broad-spectrum entry inhibitor.

## Introduction

Alphaviruses (family *Togaviridae*) are enveloped positive-strand RNA viruses. Most alphaviruses are transmitted by mosquitos and can infect a wide range of vertebrate species. In humans, chikungunya virus (CHIKV), Ross River virus (RRV), Mayaro virus (MAYV), and Sindbis virus (SINV) can cause fever, rash, headache, muscle and joint pain. Notably, in the case of CHIKV infection, arthritic symptoms can persist for months to years^1^. Eastern, Western, and Venezuelan equine encephalitis viruses (EEEV, WEEV, and VEEV) can cause fatal encephalitis. There are currently no approved drugs for treating alphavirus infections. The progress that has been made in the analysis of the structures and functions of RNA replicates and host components that are crucial for infection provides novel opportunities to inhibit multiple steps in the alphavirus infection cycle (Supplementary Fig. 1)^2–4^.

**Fig. 1:**
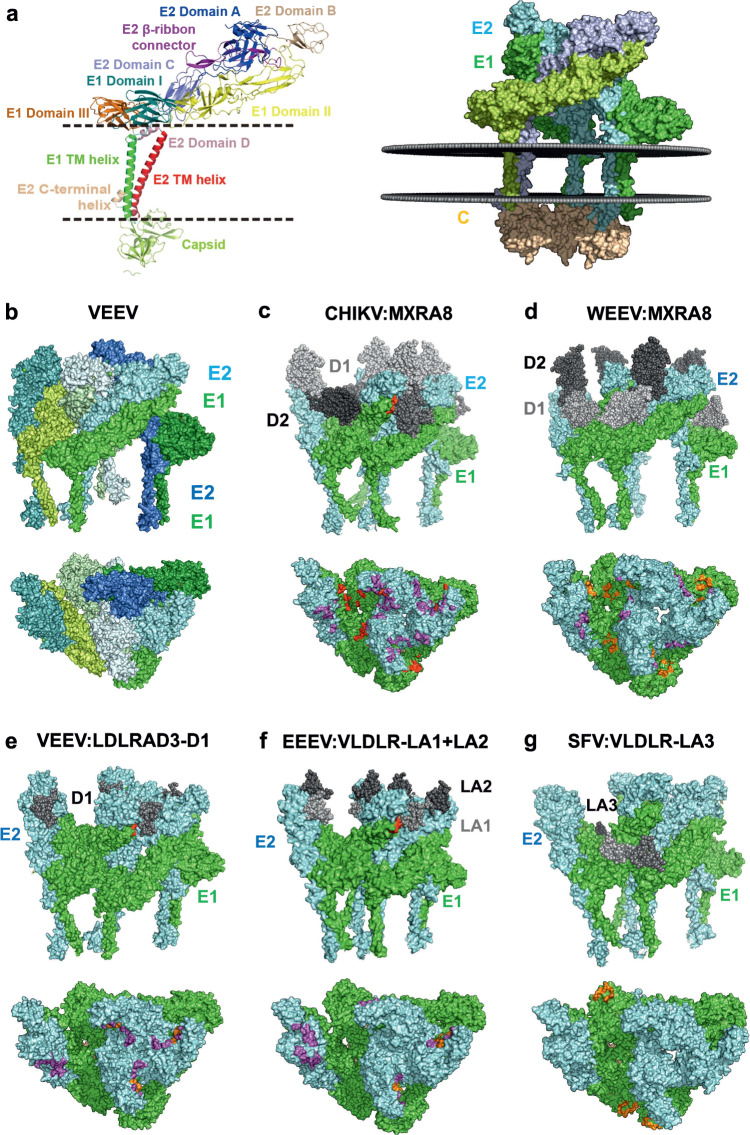
Cellular receptors of alphaviruses bind to different sites on a E1/E2 spike. **a** Left panel: Schematic presentation of domain organization of E1/E2 dimer is from Wang et al., 2022^5^. Right panel: structure of a GETV E1/E2 trimer (spike) integrated into a virtual lipid bilayer. A surface representation is shown; the capsid protein is colored in wheat, E1 in green, and E2 in cyan. One E1 and one E2 molecule, which form a heterodimer, are colored in light blue and light green, respectively. The figure was created with PyMol software using the cryo-EM structure of a GETV virion (PDB: 7FD2) and integrated into the plasma membrane using https://opm.phar.umich.edu/ppm_server3. **b**–**g** Upper parts of panels depict a surface model of one icosahedral asymmetric unit in free form (b) or bound to its receptor (c-g), which are represented using gray spheres and labeled. Amino acids in the fusion loop of E1 that interact with the receptor are shown as red spheres (in this view of the WEEV:MXRA8 complex, these residues are not visible). Lower parts of the panels display a top view of the icosahedral asymmetric unit, with amino acids of E1 and E2 interacting with the receptor shown as orange and magenta spheres, respectively. Panel (b) shows the structure of an icosahedral asymmetric unit of VEEV without a receptor, with individual monomers colored in varying shades of blue (E2) and green (E1). This figure was generated using PyMol software and data from following PDB files: 7N1I (VEEV), 6NK6 (CHIKV:MXRA8), 8DAN (WEEV:MXRA8), 7FFL (VEEV:LDLRAD3-D1), 8UFC (EEEV:VLDLR-LA1 + LA2), and 8IHP (SFV:VLDLR-LA3).

Amino acid sequence identity among the alphavirus glycoproteins is ~40% for E2 and ~ 45% for E1. Each glycoprotein consists of a short cytoplasmic tail, a transmembrane region, and a large ectodomain (Fig. 1a, b). The E1 ectodomain displays the characteristic fold of ‘class II’ viral membrane fusion proteins, consisting of three domains (I, II, and III) that are essentially folded into beta-sandwiches. The E2 ectodomain, previously assumed to be the sole receptor-binding protein, is organized in a linear arrangement of three domains (A, B, and C), each exhibiting an immunoglobulin superfamily-like fold. The E1/E2 heterodimers form trimeric spikes (Fig. 1a). 80 spikes form a regular surface lattice enclosing the viral membrane^5^. Depending on the virus species, a fraction of the spikes can remain associated with the E3 protein, which is derived from the cleavage of the E3/E2 precursor^6–8^. Exposure to low pH in the endosome induces dissociation of the E1/E2 heterodimer and surface lattice dissociation, freeing E1 to undergo a fusogenic conformational change that drives the merger of viral and endosomal membranes^9^.

The binding of a virus particle to a cell surface receptor is the first step in the infection process. Although alphaviruses have caused many outbreaks in humans and livestock and pose a serious public health threat, the nature of their receptors has been elusive. Notably, the reported alphavirus receptors, such as matrix remodeling-associated protein 8 (MXRA8), do not explain the multi-tissue and multispecies tropism of some alphaviruses. For example, in MXRA8 knockout cells, the titer of Getah virus (GETV) is only slightly decreased, and Semliki Forest virus (SFV) can even reach a titer similar to that in wild type control cells^10^. CHIKV titers in MXRA8 knockout tissues are markedly diminished, yet not abrogated, and RRV is even capable of causing disease in the absence of MXRA8^11,12^. Thus, studies conducted with the aim of discovering and characterizing new receptors used by multiple alphaviruses are essential. The recent identification of low-density lipoprotein receptors (LPRs) as functional entry factors for various alphaviruses has advanced our understanding of viral pathogenesis, tropism and evolution and is expected to contribute to the development of novel strategies for preventing and treating alphavirus infections^13–16^.

## Multiple alphaviruses use LPRs for cell entry

Among the fourteen examined alphaviruses, twelve were revealed to utilize mammalian or avian MXRA8 protein (Supplementary Table 1). Among viruses that do not use MXRA8 and exploit LPRs, VEEV utilizes LDLRAD3, while EEEV utilizes VLDLR and its close homolog ApoER2. Furthermore, many alphaviruses that utilize MXRA8 also bind to at least one LPR. In fact, GETV and SFV have been shown to exploit three different LPRs: LDLR, VLDLR, and ApoER2.

A distinguishing feature of the LPRs known to act as alphavirus receptors is the presence of multiple (3 to 8) LDL-receptor class A (LA) domain repeats (~40 amino acids) in their ectodomains. LDLR and VLDLR bind cholesterol-loaded lipoproteins via their LA domains, allowing the internalization of the receptor-ligand complexes and their release into the endosome. The multiplicity of LA repeats allows LPRs to bind several distinct protein ligands through different combinations of repeats^17^. VLDLR binds to ApoE, a component of several lipoproteins. LA1, LA2, LA3, and LA6 are important for binding VLDL. LA7 may be responsible for differences in the ligand-binding properties of VLDLR and LDLR^18^. For LDLR binding of ApoE ligand depends on LA4 and LA5, which are located at the center of the receptor^19^. LPRs are present in most, if not all, tissues and cell types and, notably, also serve as receptors for various unrelated viruses, including rhinoviruses, mosquito-transmitted vesicular stomatitis virus, and tick-transmitted Crimean-Congo hemorrhagic fever virus^20–22^. A VLDLR orthologue, called lipoprotein receptor 1, is present in mosquitoes and, when ectopically expressed in human cells, facilitates the entry of SFV and EEEV^16^.

## Alphavirus receptors: analyzing similarities and differences in binding modes

To elucidate the common regions involved in receptor binding, we used the known 3D structures of alphavirus virion: receptor complexes. Human MXRA8 (hMXRA8) establishes contacts with three different sites in each asymmetric unit of the CHIKV particle: it wraps around the distal end of one E1/E2 heterodimer (‘wrapped’ contact site) while also contacting an adjacent heterodimer of the same spike trimer (‘intraspike’ contact) and one from the neighboring spike trimer (‘interspike’ contact)^23,15^. Remarkably, the structure of duck MXRA8 (dMXRA8) bound to WEEV virions revealed an unrelated binding mode. While hMXRA8 domain 1 predominantly interacts with CHIKV E2, dMXRA8 domain 1 mainly interacts with WEEV E1 (Fig. 1c, d), and only a limited subset of E1 and E2 amino acids involved in interactions with the receptor are shared between WEEV and CHIKV^24^.

LDLRAD3 has only three LA domains in its ectodomain; from these, only the N-terminal most membrane-distal domain 1 (D1) mediates the interaction with VEEV^25,26^. VEEV interacts with D1 in a manner similar to that of CHIKV engaging with hMXRA8: both bind in the same cleft between E1 and E2 of two heterodimers (Fig. 1e). Given that D1 of LDLRAD3 is significantly smaller than the alphavirus virion binding region of MXRA8 (40 versus 270 amino acid residues), the buried surface area is also smaller (900 Å^2^ versus 2100 Å^2^)^26^, and the binding affinity of LDLRAD3-D1 for VEEV is weaker (209 nM versus 83 nM)^23^.

**Fig. 2:**
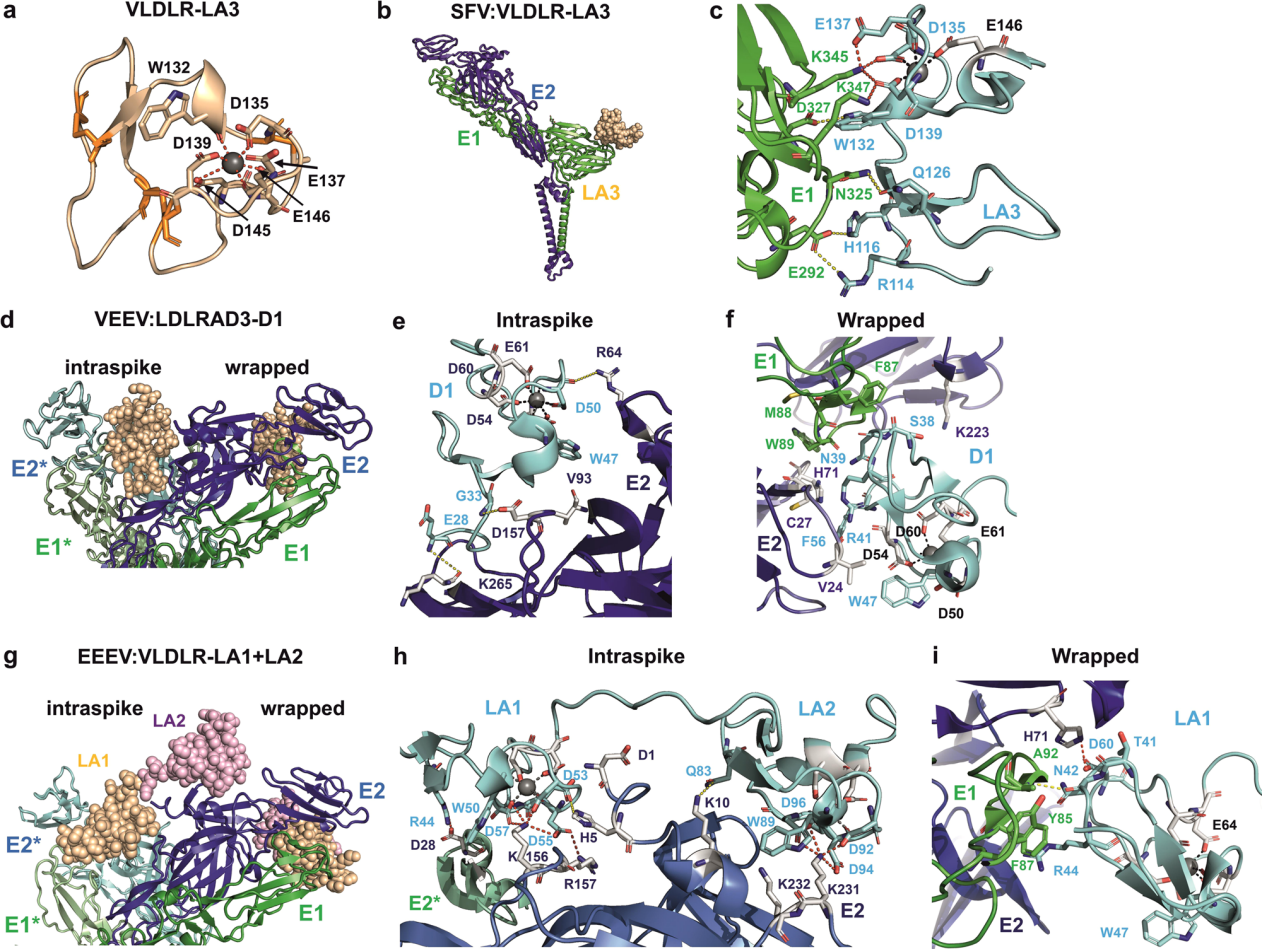
Molecular details of the interaction sites between the LPRs and E1/E2 dimers. **a** The structure of an LA domain depicted as a cartoon model of the VLDLR-LA3 domain. The Ca^2+^ ion (gray sphere) is coordinated by W132 and five acidic amino acids depicted as sticks. Note that D135, D139, D145, and E145 interact with Ca^2+^ via their side chains, while W132 and E137 interact via the main chain. The three disulfide bridges are highlighted in orange. **b** The binding site of the LA3 domain of VLDLR on an E1/E2 dimer of SFV and (**c**) the molecular details of this binding site. Salt bridges are depicted as red-stippled lines, and hydrogen bonds as yellow-stippled lines. Acidic amino acid residues coordinating the Ca^2+^ ion (D135, E137, D139) form salt bridges with two adjacent basic side chains (K345, K347) of E1. **d**–**f** Interaction of the intraspike and wrapped sites of the D1 domain of LDLRAD3 with an E1/E2 of VEEV. **d** In the intraspike site, D1 is inserted into a cleft between two heterotrimers and interacts solely with E2. In the wrapped site, D1 engages with the distal end of E1/E2 contacting both E2 and E1. **e** Molecular details of the intraspike binding site. **f** Molecular details of the wrapped binding site, which is characterized by the highlighted non-bonded contacts of residues in D1 with the fusion loop of E1 and with amino acids in E2. **g** Interaction of the binding sites of the LA1 and LA2 domains of VLDLR with two E1/E2 dimers of EEEV. **h** Molecular details of the intraspike binding site. E2* indicates the E2 protein from an adjacent heterodimer. **i** Molecular details of the wrapped binding site. This figure was generated using PyMol software using data from PDB-files 8IHP (**a**–**c**), 7N1H (d-f) and 8UFC (**g**–**i)**.

Interestingly, EEEV exhibits a different binding pattern by engaging multiple LA domains of VLDLR, specifically LA1, LA2, LA3, LA5, and LA6. Consecutive LA repeats can bind to their targets in a synergistic manner; the inter-domain flexibility of the receptor allows for alternative LA domain orientations. The structure of the EEEV:VLDLR–LA1 + LA2 complex also revealed contacts at the wrapped and intraspike binding sites observed for MXRA8 (Fig. 1f). Notably, the virions of the EEEV PE-6 strain also bind to an additional site due to the E206K substitution in the E2 protein^27^. SFV can also bind to multiple LA domains of VLDLR (LA1, LA2, LA3, and LA5). The cryo-EM structure of the SFV:VLDLR–LA3 complex shows that the domain exclusively binds to residues in E1 located at the edge of the spike (Fig. 1g)^28^. The buried surface area is the smallest (378 Å^2^)^28^; hence, the binding affinity of LA3 for SFV is the weakest (1300 nM) of all observed alphavirus: receptor interaction sites. Yet when consecutive LA domains are engaged, the binding affinity becomes stronger, up to 2 nM for the stretch of LA1 to LA6 (Supplementary Table 2)^28^. Importantly, the binding sites of VLDLR for SFV and EEEV (Fig. 2b, c, g–i) and their biochemical characteristics differ entirely (Supplementary Table 2).

Although the interactions between the LA domains and the E1/E2 spike proteins are complex and involve different regions of the spike, a closer examination at the molecular level reveals common features. The LA repeats exhibit a conserved fold that is characterized by three disulfide bonds, the coordination of one Ca^2+^ ion by the side chains of four acidic residues, by the main chain of one (often acidic) residue, and that of an aromatic tryptophan residue located on a short helix in the vicinity (Fig. 2a, Supplementary Fig. 2). In the SFV:VLDLR–LA3 complex, the binding site for the LA3 domain is located at the edge of the trimer (Fig. 2b) and involves polar interactions with E1. Three out of the five Ca^2+^-coordinating acidic residues (D135, E137, D139) form ionic interactions with two adjacent basic side chains (K345, K347) of E1. Hydrogen bonds between W132 and D327, along with other polar interactions at the N-terminus of LA3, further stabilize this complex (Fig. 2c). Notably, in the SFV:VLDLR–LA3 complex the three binding sites on a single spike are identical, which sets it apart from the other complexes.

Two different binding sites are present in the VEEV:LDLRAD3–D1 complex, as well as in the EEEV:VLDLR–LA1 + LA2 complex. One corresponds to the wrapped site also targeted by MXRA8, located at the distal edge of one heterodimer, where the receptor interacts with both E2 and E1. At the intraspike site, the LA domains are inserted into the cleft between two E2/E1 heterodimers from the same spike, and only the E2 protein is involved in binding (Fig. 2d, g). The interactions at the intraspike site are mainly governed by ionic bonds between Ca^2+^-coordinating acidic amino acids and basic residues in E2. At the intraspike site of the VEEV:LDLRAD3–D1 complex, D50 interacts with R64 via its main chain, and W47 interacts with V93 of E2. While further polar interactions (D157 and K265 of E2 interact with the G33 and E28 residues of D1, respectively) stabilize binding, no interaction of the receptor with E2 belonging to another heterodimer was observed (Fig. 2e). In the EEEV:VLDLR–LA1 + LA2 complex, the interactions with Ca^2+^-coordinating acidic residues are more prominent. In both LA1 and LA2 domains, two acidic residues (D55 and D57 in LA1; D94 and D96 in LA2) form ionic bonds with two (K156 and R157) or a single basic residue (K231) in E2. W50 and W89 also engage with the basic residues of E2 (K156 and K232, respectively). The interaction is further strengthened by two additional salt bridges: R44 interacts with D28, which is located in E2 of the neighboring heterodimer, and D53, located near the Ca^2+^-binding site in LA1, interacts with K156 in E2 via its side chain and with H5 in E2 via its main chain (Fig. 2h).

The wrapped binding sites of both the VEEV:LDLRAD3–D1 and EEEV:VLDLR–LA1 + LA2 complexes are characterized by the involvement of the fusion loop of E1 in the interactions, in particular involving non-polar contacts with aromatic amino acids (Fig. 2f, i). Similarly, the fusion loop also plays a significant role in the binding of MXRA8 to CHIKV and WEEV (Fig. 1c, d). Within the LA domain, the interactions occur on a different surface, thus the Ca^2+^-coordinating residues do not participate in spike-receptor interactions (Fig. 2f, i). In general, the wrapped binding sites are characterized by few polar interactions. Only the EEEV:VLDLR–LA1 + LA2 complex exhibits an ionic bond between D60 in LA1 and H71 in E2 and a hydrogen bond between N42 in LA1 and the main chain of A92 in E1 (Fig. 2i). In this complex, LA2 does not participate in the binding of the wrapped site; instead, the domain protrudes from the surface of the trimer (Fig. 2g).

Very recently, two studies identified LDLR as a receptor for GETV, SFV, Bebaru virus (BEBV), RRV, EEEV, and WEEV^13,14^. To date, no structural information exists on the virion:LDLR complex for any of these viruses. Functional analysis revealed that GETV binds to LDLR with relatively high affinity, and the deletion of (or mutations in) LA4 and LA5 domains prevent or decrease viral cell entry^13^. In contrast, EEEV and SFV particles were shown to interact with LA3 and LA4, respectively, displaying low binding affinity^14^. These data suggest that the modes used by virions of different alphaviruses for binding to LDLR are also variable.

## The lack of a distinct receptor-binding domain (RBD) has implications for alphavirus transmission

Our analysis suggested that alphavirus virion spikes do not have a distinct RBD; instead, the receptor binding sites display significant variability across any given spike:receptor complex. The regions of the LPRs that bind alphaviruses are similar to the key regions involved in the binding of their physiological ligands, and a single domain of the receptor can interact with multiple sites in one spike (Figs. 1,  2). This complex diversity underscores a fundamental distinction in the receptor recognition mechanisms employed by alphaviruses compared to those employed by coronaviruses. Virions of coronaviruses are distinguished by elongated surface projections that represent homotrimeric spike (S) protein. This surface topology likely has played a crucial role in the evolution of a distinct RBD that is located at the apices of these surface proteins and serves as the primary point of interaction between the virus particle and host cell. In contrast, similar to many arboviruses, alphavirus virions exhibit a comparatively smoother surface and lack a predisposed region for RBD development. Consequently, alphaviruses possess multiple receptor binding sites that are distributed across the surfaces of virion glycoproteins, enabling them to interact with various cellular receptors. This versatility in receptor(s) binding may account for the ability of alphaviruses to infect evolutionarily diverse hosts. This ecological niche imposes specific constraints on the molecular evolution of alphaviruses. Consequently, the acquisition of mutations that facilitate host switching appears to be infrequent in alphaviruses compared to coronaviruses, which have demonstrated more frequent interspecies transmission events. In these cases, mutations enhancing receptor binding in a new host are more likely to be positively selected, facilitating rapid adaptation to novel hosts.

The critical virion-binding region of LDLRAD3 is highly conserved (Supplementary Fig. 2a)^15^. This finding is consistent with the enzootic cycle of VEEV, in which the virus circulates between mosquitoes and rodents, with accidental spillover into bats^29^ and horses. Similarly, VLDLRs in humans, mice, horses, and birds show high conservation of the key residues recognized by SFV or EEEV (Supplementary Fig. 2b)^27,28^. LDLR sequence alignments revealed that residues shown to have a significant impact on GETV binding are highly conserved in mammals but are quite different in avian LDLRs (Supplementary Fig. 2c). This finding is consistent with the observation that GETV infects mainly mammals. A similar trend was observed for the residues recognized by EEEV. Ma and colleagues identified three amino acid groups (D112 and E113; F126 and V127; D136 to A141) that significantly impact the ability of the LA3 domain of human LDLR to support the infection of chimeric SINV harboring glycoproteins of EEEV^14^. Mammalian and avian LDLRs were found to harbor differences among these groups (E/D113Q, V127A/L and A141 R/L; Supplementary Fig. 2c). In contrast to GETV, the natural amplification hosts of EEEV are songbirds^30^ and only a few mammalian species can be naturally infected with EEEV. Therefore, the possibility that EEEV may prefer to use avian LDLR as an entry receptor cannot be excluded. Whether different alphaviruses might have adapted to use mammalian, avian, and possibly reptilian LPRs during evolution deserves further investigation.

Despite the diversity of receptor binding sites, they also exhibit conserved features. At least three of the Ca^2+^-coordinating amino acids (tryptophan and the following two acidic residues) are involved in interactions with the binding sites of SFV virions and with intraspike sites of VEEV and EEEV (Supplementary Figs. 2d, 3). The functional importance of these residues is illustrated by findings indicating that mutations of tryptophan and at least one of the following two acidic residues strongly reduce GETV infection in cells expressing these variants of LDLR^13^. Amino acids implicated in binding at the wrapped site also exhibit shared sequence characteristics, particularly residues positioned between the second and third cysteine residue of the LA domain (sequence NGRC) (Supplementary Fig. 2e). Given the high conservation and indispensable role of Ca^2+^-coordinating amino acids in the LA domains, it is plausible that alphaviruses underwent early evolutionary adaptations to utilize LPRs as key mediators of cellular entry.

The existence of diverse modes of spike and receptor interactions increases the number of possible ways for alphaviruses to bind a receptor or different receptors. A synergistic binding mode could facilitate the attachment of a virus to consecutive LA domains. The binding of multiple LA repeats also increases the overall binding efficiency, which, coupled with the flexibility that allows for variable orientations of the individual LA domains, may also enable alphaviruses to bind to LPRs from multiple species. This may serve as a prerequisite for host- and vector switches and thus compensate for the lack of RBDs that can easily acquire adaptive mutations. Notably, the expression of one receptor can also compensate for the lack of other receptors, implying that finding even one suitable receptor is likely sufficient for an alphavirus to infect a new host^13^. Once this occurs, the existence of various binding modes may additionally facilitate the subsequent adaptation of alphaviruses to new hosts, vectors, and/or cell types. For example, recent studies have shown that expression of murine MXRA8 facilitates RRV infection, whereas avian MXRA8 expression does not^24^. However, RRV has been isolated from passerine birds, indicating that they can act as amplifying hosts^31^. It was also shown that mutation of residue 218 of E2 of RRV that increases the binding of virions to heparan sulfate enables RRV to infect avian-derived cell cultures^32^. Therefore, we speculate that in nature, RRV may be undergoing adaptive evolution in birds due to its ability to bind heparan sulfate and utilize LDLR as an entry receptor.

The amino acid sequence identity between ApoER2 and LDLR is 49%, and that between ApoER2 and VLDLR is ~47%. High sequence identity likely allows alphaviruses to infect tissues and organs where only some of these receptors are present. Furthermore, the close relationship between these three proteins may play a role in the ability of alphaviruses to infect new hosts, making virus recombination a possibility. WEEV, Highlands J virus, and Fort Morgan virus underwent an ancestral recombination event involving EEEV-like and SINV-like viruses^33^. Similar events may also occur in the future. For example, simultaneous amplification of CHIKV and GETV has been observed in pangolins in Guangxi Province, China, indicating cotransmission of these viruses^34^. Because GETV possesses diverse receptor binding properties, it may, through coinfection and recombination events, relatively easily overcome existing host range barriers and thus represent a potential risk to public health^35^.

## Perspectives for the development of broad-spectrum entry inhibitors

**Fig. 3:**
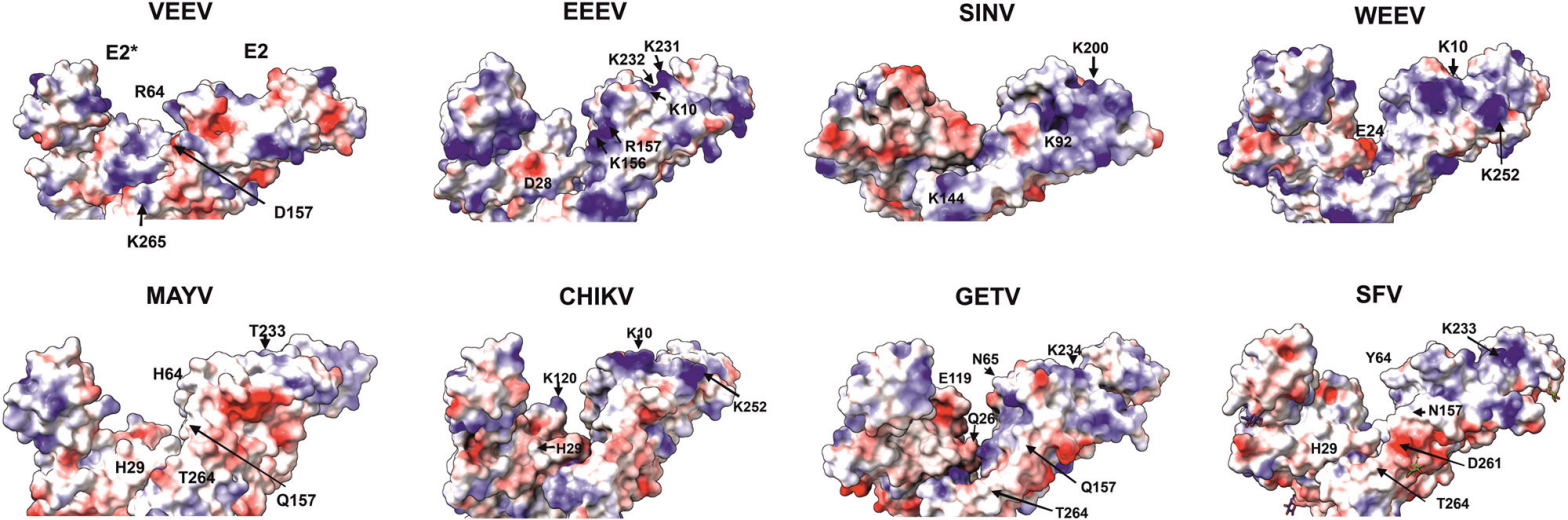
Electrostatic surface potential of the cleft between two E1/E2 dimers of different alphaviruses. Only two E2 subunits are shown. In VEEV and EEEV the residues forming polar bonds with LDLRAD3-D1 and VLDLR-LA1 + LA2, respectively, are indicated. Amino acids at a similar position and prominent charged residues are indicated on the other structures. The figure was created with the built-in ABPS function of ChimeraX using the PDB files mentioned above and 7KO8 (MAYV), 6IMM (SINV), and 7WC2 (GETV).

The ability of alphaviruses to use alternative receptors and exploit different modes to interact with them has been a challenge for the development of entry inhibitors. On the other hand, our analysis revealed that the receptor binding sites (the wrapped site and the intraspike site) are shared by different receptors. Soluble proteins, which correspond to the extracellular domains of alphavirus receptors, can reduce disease progression in vivo^14,16^. Furthermore, a single LDLR decoy reduces the pathogenicity of GETV, which can also use VLDLR, ApoER2, and MXRA8^13^, and a chimeric avian-mammalian MXRA8 decoy receptor has been shown to neutralize infections caused by alphaviruses from distinct antigenic groups^24^. These data indicate that a sufficiently high-affinity interaction with one decoy receptor might block virion interactions with multiple receptors as long as the same binding site is used. To prevent infection, a decoy likely needs to occupy a large proportion of the up to 240 receptor binding sites on each virus particle. This may require the use of high concentrations of the inhibitor, which could potentially result in cytotoxic effects. Thus, mimetics of the receptor domains that fit better into the individual receptor binding sites should be used. Alternatively, these compounds could transiently interact with spikes, triggering irreversible conformational changes that inactivate virus particles. Furthermore, it is possible that a compound that is compatible with the intraspike site and interacts with the positively charged amino acids that contact the receptor could inhibit a wide range of alphaviruses. A comparative analysis of the electrostatic surface potential in the cleft between the E2 proteins of the two heterodimers was performed. The spikes of alphaviruses were found to exhibit variations in dimension and shape of the cleft, and in its surface charges (Fig. 3), implying that the effectiveness of such a strategy is likely limited. The most conserved elements of LPRs are Ca^2+^ coordinating motifs that are also required for the binding of alphavirus virions (Supplementary Fig. 2d, e). Hence, the targeting of these motifs offers the possibility to develop broad-spectrum antivirals. Finally, the physiological functions of LDLRs and VLDLRs may be advantageous as well. Because of their role in the transport of a variety of lipoproteins, natural and synthetic LDL carriers, which exploit mechanisms of native LDL uptake and act as ‘eat-me’ signals, have been widely used for the targeted delivery of drugs developed to treat glioblastoma and other diseases^36^. It is conceivable that modified apolipoproteins and lipoproteins, composed of such molecules and capable of competitively binding to the receptor, may also have antiviral functions.

Taken together, these findings indicate that the lack of a unique binding site on the alphavirus surface makes the development of broad-spectrum inhibitors challenging. Yet, the observation that the same wrapped and intraspike sites, initially observed in the CHIKV–MXRA8 complex, are used by most of LPRs suggests that a single inhibitor could efficiently block the interaction with multiple receptors. Indeed, the results obtained using receptor and ligand decoys are encouraging. Further fundamental research on the binding conditions and modes of different alphaviruses to their receptors can provide useful starting points for the development of such inhibitors.

### Supplementary information

Supplementary Information
