# Supplementary material for: LDL receptor in alphavirus entry: structural analysis and implications for antiviral therapy
Source: Nat Commun. 2024 Jun 8;15:4906. doi: 10.1038/s41467-024-49301-1 (PMC11162471; doi:10.1038/s41467-024-49301-1)
Supplement: Supplementary file 1 — Supplementary Information [file 41467_2024_49301_MOESM1_ESM.pdf]

Supplementary Fig. 1

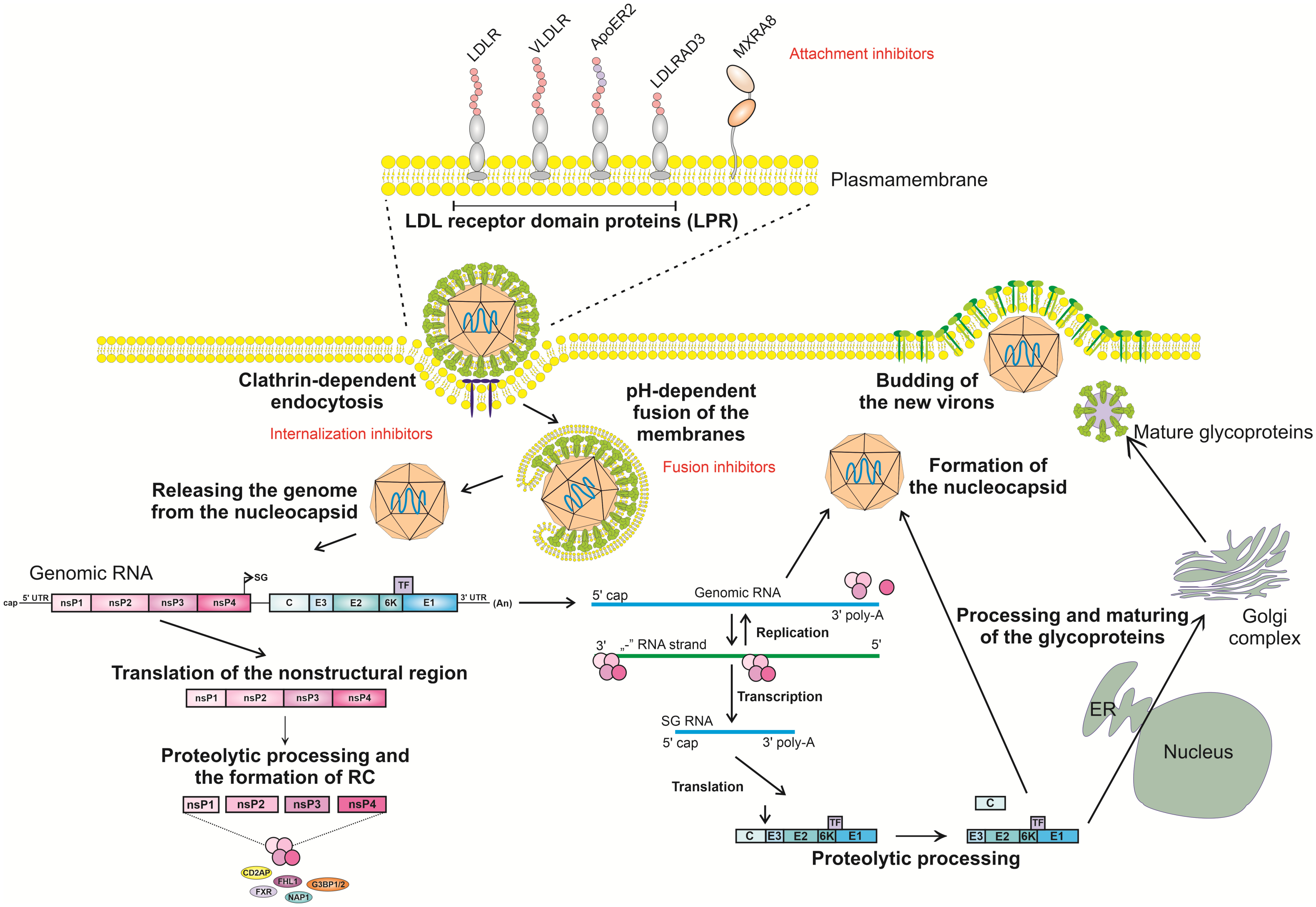

Supplementary Fig. 1 Alphavirus infection cycle offers targets for development of broad-spectrum inhibitors. Schematics of virus genome organization and infection cycle are provided. Five receptors, discussed in this analysis, are indicated. Broad spectrum inhibitors can be used to inhibit the steps of an entry process of the virus (indicated) or to interfere with subsequent RNA replicase formation and its activities (not covered in this analysis).

Supplementary Fig. 2

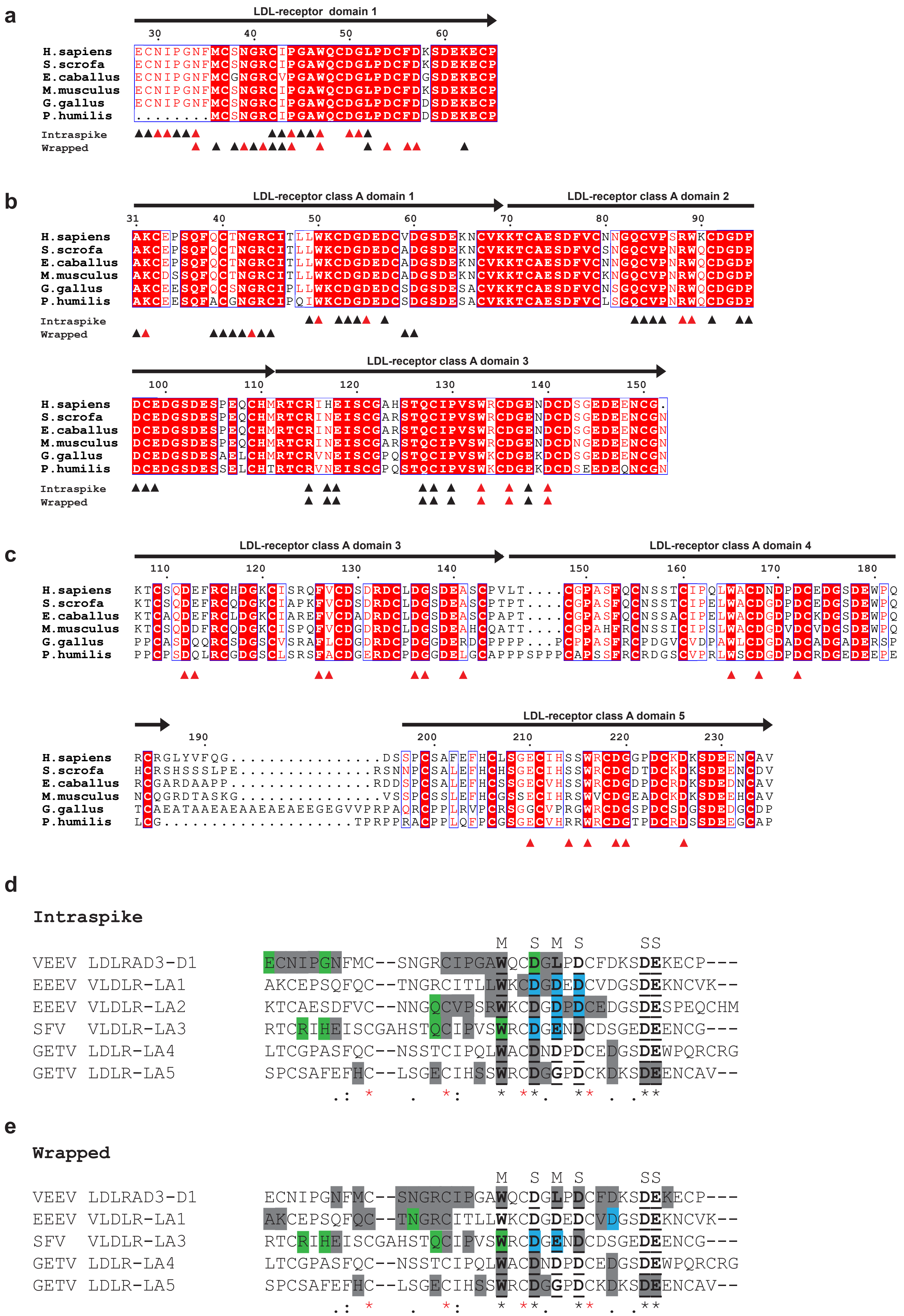

Supplementary Fig. 2 Comparison of E1/E2 binding sites in LPRs. (**a**-**c**) The LA-receptor domains of LDLRAD3 (**a**), VLDLR (**b**), and LDLR (**c**) from human (*Homo sapiens*, NM_174902.4, NM_003383.5, NM_000527.5), pig (*Sus scrofa*, XM_021083183.1, NM_001199890.1, NM_001206354.2), horse (*Equus caballus*, XM_023653877.1, XM_023627269.1, NM_001206354.2), mouse (*Mus musculus*, NM_178886.3, NM_013703.2, NM_010700.3), chicken (*Gallus gallus*, XM_040701387.2, NM_205229.2, NM_204452.1), and songbird ground tit (*Pseudopodoces humilis*, XM_014252176.1, XM_005522700.2, XM_014262128.1), shown to interact with alphavirus virions, were aligned using ClustalW (http://www.genome.jp/tools-bin/clustalw). The numbering of amino acid residues in these domains is based either on the sequence of human (**a**, **b**) or pig (**c**) proteins. Completely conserved residues are shown in white on a red background; highly conserved residues are boxed and shown in red. Triangles represent residues that are shown to form contacts with E1/E2 of different alphaviruses. Red triangles represent residues that have been shown to affect alphavirus infection. (**d-e**) The amino acid sequences of the E1/E2 binding domains of LPRs from mouse (*Mus musculus*) were aligned with ClustalAl Omega (https://www.ebi.ac.uk/jdispatcher/msa/clustalo). Residues that are in contact with E1/E2 of the indicated virus are highlighted as follows: grey, non-polar contacts; green, hydrogen bonds; blue, ionic bonds. Residues coordinating the Ca^{2+} ion are denoted in bold and underlined. The letters in the top lines indicate whether the side chain (S) or main chain (M) of the amino acid in the respective column interacts with Ca^{2+}. Note that SFV has a single binding site that does not correspond to an intraspike or wrapped site. While the structure of GETV with the receptor domains of LDLR remains unresolved, substitutions in the highlighted residues have been shown to reduce virus infection in cells expressing corresponding LDLR mutants. “ * ” represent conserved Ca^{2+} ion binding motif. “ Red * ” represent completely conserved cysteine resides. “:” represent residues with very similar properties and “.” represent residues with similar properties.

Supplementary Fig. 3

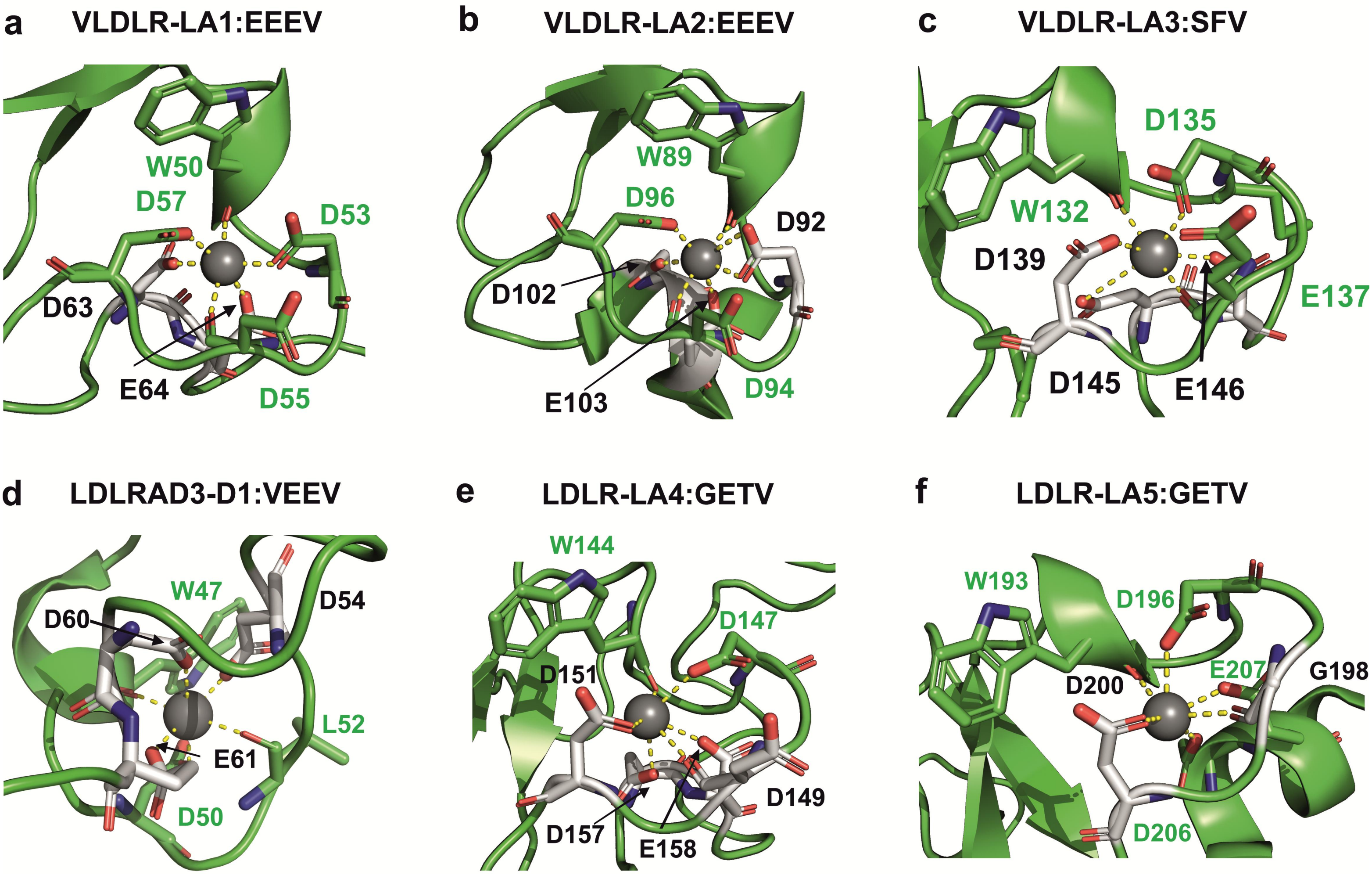

Supplementary Fig. 3. Ca^{2+} binding sites in D1 and LA domains are involved in the binding of indicated viruses. Ca^{2+} binding sites in of LPRs are crucial for interaction with E1/E2 of EEEV (**a**, **b**), SFV (**c**), VEEV (**d**), GETV (**e**, **f**). Residues contacting Ca^{2+} are depicted as sticks. Green sticks labelled in green letters represent residues that also interact with E1/E2. This visualization highlights the dual role of these residues in the Ca^{2+} binding and in interactions with E1/E2 glycoproteins. Figures were created with PyMol software from PDB-files 8UFC (**a**-**b**), 8IHP (**c**) and 7FFL (**d**). **e** and **f** were created from PDB-file 2LGP using experimental data.

Supplementary Table 1. Receptors used by different alphaviruses.

Virus	LDLR	LDLRAD3	VLDLR	ApoER2	MXRA8
GETV	+	-	+	+	+[a]
SFV	+	-	+	+	+[a]
BEBV	+	-	n.a.	n.a.	+[a]
RRV	+	-	n.a.	n.a.	+[a]
VEEV	-	+	-	-	-
WEEV	+	-	-	-	+[b]
EEEV	+	-	+	+	-
CHIKV	-	-	-	-	+[a]
MAYV	-	-	n.a.	n.a.	+[a]
MIDV	-	-	n.a.	n.a.	+[a]
SINV[#]	-	-	+	+	+[b]
WHAV	n.a.	n.a.	n.a.	n.a.	+[b]
ONNV	n.a.	n.a.	n.a.	n.a.	+[a]
BFV	n.a.	n.a.	n.a.	n.a.	+[a]
Reference	[1, 2]	[3]	[4]	[4]	[1, 5, 6]

MIDV – Middelburg virus; ONNV – o'nyong'nyong virus, BFV – Barmah Forest virus, WHAV – Whataroa virus. Other abbreviations are provided in the main text.

+, serves as receptor; -, does not serve as receptor; n.a., not analyzed; +[a], uses mammalian MXRA8; +[b], uses avian MXRA8.

[#] SINV is also shown to use natural resistance-associated macrophage protein (NRAMP) as a receptor[7].

Supplementary Table 2. Biochemical characteristics of known alphavirus particle:LPR complexes.

Complex	Number of interacting residues	Number of interactions	Ionic bonds	H-bonds	Interacting surface (Å)	Affinity K_d (nM)	Remarks
VEEV with domain 1 of LDLRAD3	15-19 (domain 1)<br>14-17 (E2)<br>2-3 (E1)	72-99	2	2-3	900-1085	D1: 209<br>D1+D2: 50	Polar interaction
Wrapped with E2	3-4 (domain 1)<br>5-7 (E2)	18-23	0	0-1	315-320		
Wrapped with E1	3-4 (domain 1)<br>2-3 (E1)	7-13	0	0	180-205		
Intraspike with E2	9-11 (domain 1)<br>9-10 (E2)	47-63	2	2-3	550-560		
SFV with LA3 of VLDLR	8-10 (LA3)<br>8-9 (E2)	42-53	5-7	2-7	350-378	LA3: 1300<br>LA1-6: 2	Polar interaction
EEEV with LA1+LA2 of VLDLR	31 (LA1)<br>3 (linker)<br>22 (LA2)<br>31 (E2)<br>12 (E1)	2	8	2	~1100	LA1-2: 100/500<br>LA1-3: 34<br>LA3-5: 32	Mostly non-polar interaction
Wrapped with E2	10 (LA1)<br>7 (E2)	?	5	?			
Wrapped With E1	10 (LA1)<br>12 (E1)	?	0	?			
Intraspike with E2	11 (LA1)<br>3 (linker)<br>22 (LA2)<br>24 (E2)	?	3 with LA2	?			

The numbers were generated with PDBsum from PDB file 7FFF (VEEV with LDLRAD3), 8IHP (SFV with VLDLR), and 8UFC (EEEV with VLDLR).

The bio-layer interferometry affinity measurements with virus-like particles and the indicated domains are from the papers describing the respective structures.

"Wrapped" indicates that the respective domain interacts with E1 and E2 in the same heterodimer and "intraspike" with E2 in an adjacent heterotrimer of the same spike.

References

1. Zhai X*, et al.* LDLR is used as a cell entry receptor by multiple alphaviruses. *Nature Communications* **15**, (2024).
2. Ma H*, et al.* The low-density lipoprotein receptor promotes infection of multiple encephalitic alphaviruses. *Nat Commun* **15**, 246 (2024).
3. Ma H*, et al.* LDLRAD3 is a receptor for Venezuelan equine encephalitis virus. *Nature* **588**, 308-314 (2020).
4. Clark LE*, et al.* VLDLR and ApoER2 are receptors for multiple alphaviruses. *Nature* **602**, 475-480 (2022).
5. Zimmerman O*, et al.* Vertebrate-class-specific binding modes of the alphavirus receptor MXRA8. *Cell* **186**, 4818-4833 e4825 (2023).
6. Zhang R*, et al.* Mxra8 is a receptor for multiple arthritogenic alphaviruses. *Nature* **557**, 570-574 (2018).
7. Rose PP*, et al.* Natural Resistance-Associated Macrophage Protein Is a Cellular Receptor for Sindbis Virus in Both Insect and Mammalian Hosts. *Cell Host Microbe* **10**, 97-104 (2011).
